# *Astragalus mongholicus* Bunge (Fabaceae): Bioactive Compounds and Potential Therapeutic Mechanisms Against Alzheimer’s Disease

**DOI:** 10.3389/fphar.2022.924429

**Published:** 2022-06-28

**Authors:** Qianyu Dong, Zhen Li, Qian Zhang, Yueyu Hu, Huazheng Liang, Lize Xiong

**Affiliations:** ^1^ Shanghai Key Laboratory of Anesthesiology and Brain Functional Modulation, Shanghai, China; ^2^ Clinical Research Center for Anesthesiology and Perioperative Medicine, Shanghai Fourth People’s Hospital, School of Medicine, Tongji University, Shanghai, China; ^3^ Translational Research Institute of Brain and Brain-Like Intelligence, Shanghai Fourth People’s Hospital, School of Medicine, Tongji University, Shanghai, China; ^4^ Department of Anesthesiology and Perioperative Medicine, Shanghai Fourth People’s Hospital, School of Medicine, Tongji University, Shanghai, China; ^5^ Department of Neurology, Shanghai Fourth People’s Hospital, School of Medicine, Tongji University, Shanghai, China

**Keywords:** *Astragali radix*, alzheimer’s disease, traditional Chinese medicine, bioactive compound, molecular mechanism

## Abstract

*Astragalus mongholicus* Bunge (Fabaceae) (also known as *Astragali radix*-AR), a widely used herb by Traditional Chinese Medicine practitioners, possesses a wide range of pharmacological effects, and has been used to treat Alzheimer’s disease (AD) historically. Its bioactive compounds are categorized into four families: saponins, flavonoids, polysaccharides, and others. AR’s bioactive compounds are effective in managing AD through a variety of mechanisms, including inhibiting Aβ production, aggregation and tau hyperphosphorylation, protecting neurons against oxidative stress, neuroinflammation and apoptosis, promoting neural stem cell proliferation and differentiation and ameliorating mitochondrial dysfunction. This review aims to shed light upon the chemical constituents of AR and the mechanisms underlying the therapeutic effect of each compound in manging AD. Also presented are clinical studies which reported successful management of AD with AR and other herbs. These will be helpful for drug development and clinical application of AR to treat AD.

## 1 Introduction

Alzheimer’s disease (AD), the most common neurodegenerative disease, is the dominant cause of dementia and associated with aging and other factors (Scheltens et al., 2016; Long and Holtzman, 2019). According to statistics from Alzheimer’s Disease International, Worldwide there are over 50 million people suffering from dementia in 2018, and 152 million by 2050. Among them, approximately 60–70% are inflicted by AD (Zhang et al., 2022). As the aging population increases, AD poses a great challenge to the society in terms of medical resources and economic burden (Alzheimer’s Association, 2020).

AD is pathologically characterized by two hallmarks: extracellular senile plaques (SPs) formed by amyloid-β (Aβ) deposited in the extracellular space, and neurofibrillary tangles caused by the aggregation of intracellular hyperphosphorylated tau proteins (Karran et al., 2011; Knopman et al., 2021). Aβ plaques and neurofibrillary tangles play an important role in eliciting the dysfunction of synapses and associated neuronal death, leading to typical symptoms of AD, such as cognitive impairment, memory loss, and so on (Hong et al., 2016). A number of hypotheses have emerged to illustrate the pathogenesis of AD, mainly the amyloid cascade hypothesis (Savelieff et al., 2013; Butterfield and Halliwell, 2019; Hampel et al., 2021), tau protein hypothesis (Arnsten et al., 2021), metal ion disorder hypothesis (Savelieff et al., 2013), oxidative stress hypothesis (Butterfield and Halliwell, 2019), and cholinergic hypothesis (Hampel et al., 2018), etc. However, clinical trials so far have failed to demonstrate the efficacy of various therapeutics targeting the above mechanisms (Breijyeh and Karaman, 2020). Currently, there are only 5 medications approved by FDA to treat AD, all with limited effects; which encourages us to find an answer in the traditional medicine.

Traditional Chinese Medicine (TCM) has been practiced in China for thousands of years. It has its own unique theory of the human body, including its composition, connections between organs and recipes that target dysfunctioning organs. *Astragalus mongholicus* Bunge (Fabaceae), also known as *Astragali radix* (AR) or *Huang qi*, is a popular herbal medicine in China, originally recorded in *Shennong’s Classic of Materia Medica*. According to the Pharmacopoeia of the People’s Republic of China, AR is the dry root of *Astragalus membranaceus* (Fisch.) Bge. or *Astragalus membranaceus* (Fisch.) Bge. *var. mongholicus* (Bge.) Hsiao (Fabaceae) (Li et al., 2014). Modern Chinese pharmacological studies indicate that AR is mainly used as a blood-nourishing, diuretic, tonic, expectorant, and detoxicating agent (Fu et al., 2014). Additionally, this herb exerts anti-aging, antioxidant, anti-inflammatory, immunomodulating and antiviral effects, among others (Ryu et al., 2008; Shahzad et al., 2016; Wu and Hu, 2020; Gong et al., 2021).

Nowadays, there is an increasing number of studies reporting the therapeutic effect of AR on AD, attributed to the large number of its chemical constituents. Many of them have been found to possess pharmacological activities, such as improving memory and cognitive function (Sun et al., 2020). They could potentially halt neurodegeneration through multiple components, multiple pathways, and multiple targets. So far, over 100 compounds have been isolated and authenticated from AR. They can be structurally categorized into four types: saponins, flavonoids, polysaccharides, and others (Gong et al., 2018). A variety of saponins have been isolated from AR, including astragaloside I-VIII, acetylastragaloside I, isoastragaloside I, isoastragaloside II, isoastragaloside IV, soyasaponin I, soyasaponin II, etc. Studies have shown that astragaloside Ⅳ (AS-IV) and cycloastragenol have extensive biological activities, and some of them are associated with AD pathogenesis. Astragalus polysaccharide (APS), an important active macromolecule of AR, is mainly classified into two groups: dextran and heteropolysaccharides (Liu P et al., 2017). They exert multifarious effects, such as anti-inflammatory, antioxidant, immunomodulating activities, and potential therapeutic potency for neurologic diseases (Wang et al., 2013; Zhou et al., 2017; Zhang et al., 2020). Till now, about 40 subtypes of flavonoids have been isolated and authenticated from AR, including quercetin, formononetin, kaempferol, rhamnetin, isorhamnetin, genistein, verbascoflavones, etc (Yang et al., 2020), but only a portion of them are absorbable in the digestive tract. Among them, the primary active ingredients are isoflavones and isoflavone glycosides, which possess antioxidant activities. Administration of quercetin in early-middle stage of AD pathology could attenuate cognitive impairment through increasing Aβ clearance and diminishing astrogliosis (Lu et al., 2018). Additionally, there are other extracts isolated from AR, including β-sitosterol, chlorogenic acid, caffeic acid and so on.

In this review, we will introduce the major components of AR—saponins, flavonoids, and polysaccharides regarding their preventive and protective effects on AD, as well as the underlying mechanisms. This might accelerate the discovery of novel target-specific drugs with fewer sideeffects and higher therapeutic efficacy.

## 2 Pharmacokinetics of *Astragali radix* Extracts

A total of 26 orally available compounds were identified from the AR extract using a computational chemistry prediction method, including 12 flavonoids, 5 phenolic acids, 5 nitrogen-containing compounds, 3 lignanoids and 1 coumarin. Twenty one of them were identified in *in vitro* and *in vivo* experiements, using HPLC- diode array detection-electrospray ion trap tandem mass spectrometry. These absorbable compounds include calycosin, formononetin, (6aR, 11aR)-3-hydroxy-9,10-dimethoxypterocarpan, 7,2′-dihydroxy-3′,4′-dimethoxyisoflavan, 7,2′-dihyoxy-3′,4′-dimethoxyisoflavan-7-O-β-D-glucoside -6″-O-malonate, (6aR, 11aR)-3-hydroxy-9,10-dimethoxypterocarpan-3-O-β-D-glucoside. Except calycosin which was metabolized into calycosin sulfate, these absorbable compounds were glucuronized *in vivo* during the metabolism (Xu et al., 2006).

Shi et al. (2015) tested the pharmacokinetics of the AR water extract in male rat plasma using ultra-performance liquid chromatography–tandem mass spectrometry (UPLC–MS/MS). After oral administration of the extract, 8 compounds were successfully detected in the rat plasma. Most of them were isoflavonoids and their metabolites. The concentration of these isoflavonoids, including formononetin, ononin and calycosin-7-β-glucoside, was much lower than that of their metabolites in the rat plasma. A double-peak elimination phase was observed for the majority of these compounds, except AS-IV. The mean plasma concentration-time curves showed that the values of t_max_ of all detected compounds were less than 1 h, demonstrating that they could be quickly absorbed in the gastrointestinal tract of rats.

Although daidzein could not be detected in the extract, its metabolite daidzein-7-glucuronide was found in the plasma, demonstrating the widespread presence of biotransformation between these isoflavonoids *in vivo.*

It was also shown that Calycosin-7-β-glucoside and ononin had the lowest C_max_, suggesting that they were rapidly transformed into their glucuronides. In fact, the majority of these compounds had a t_1/2_ of 3–5 h.

Although AS-IV is relatively abundant in the herb, its concentration in the plasma was lower than that of others. It might have been deglycosylated by bacteria in the intestine and eliminated from the body, as five of its metabolites were not detected in the plasma.

Additionally, Liu X et al. (2014) reported that six bioactive compounds were detected in the rat plasma after oral administration of 95% ethanol extract of AR. These included AS-IV, AS-II, formononetin, ononin, calycosin-7-β-glucoside and calycosin. Their presence and purity were normally used for quality control of AR. Using UPLC-MS/MS, pharmacokinetics of these six compounds were simulataneously studied. Among them, both AS-II and IV fitted into a two-compartment model in the rat plasma. In general, the t_1/2_ parameters of flavonoids and glycosides were shorter than their aglucones, respectively. Among these six compounds, formononetin was assimilated and cleared at the slowest speed. These suggested that the main compounds detected in the plasma could be rapidly absorbed in the gastrointestinal tract after oral administration of extracts from AR.

## 3 Clinical Use of *Astragali radix* for Alzheimer’s Disease Management

Clinical TCM studies have shown that a myriad of herbs are able to halt the progression of AD. A large number of formulations have been developed based on the individual condition of each patient and the pharmacological interactions of these herbs and most included AR as a key ingredient, as follows.

### 3.1 The Buyang Huanwu Decoction

Is a traditional prescription for the treatment of neurodegenerative disorders like AD with confirmed clinical efficacy (Liu et al., 2019; Gao et al., 2021). According to the network pharmacology analysis, AR is the main pharmacological ingredient in BHD (Gao et al., 2021). Liu et al. (2019) showed that BHD suppressed the increase of receptors for advanced glycation endproducts (RAGE), NF-κBP65, inflammatory cytokines-ICAM-1 and VCAM-1, due to the addition of Aβ_25–35_ to the cultured brain microvessel endothelial cells. In the meantime, levels of lipoprotein receptor-related protein 1 (LRP1), a key protein for Aβ clearance, and ApoE were decreased in this cell culture model, but were reversed by BHD.

### 3.2 The Naoxintong Capsule

Derived from the BHD, is a traditional patented Chinese medicine consisting of 16 herbs. It is mainly used for the treatment of cerebrovascular and cardiovascular diseases, including vascular dementia, ischemic cerebrovascular diseases, coronary heart diseases, etc. A study on the APP/PS1 double transgenic mouse model reported that NXTC could downregulate levels of Aβ, p-tau, apoptosis, and inflammatory cytokines, like IL-6, IL-1β and TNF-α through suppressing the toll like receptor 4/NF-κB/IL-1β signaling pathway. As a result, NXTC attenuated spatial memory deficit and cognitive decline. In HT-22 cells, it has been shown to block L-glutamic acid-induced production of reactive oxygen species (ROS) (Wang et al., 2021b).

### 3.3 The Buyuan Congnao decoction

Is also composed of AR mainly, Polygala *senega* L. (Polygalaceae), *Reynoutria multiflora* (Thunb.) *Moldenke* (Polygonaceae), *Acorus calamus* var. angustatus Besser (Acoraceae), *Glycyrrhiza glabra* L. (Fabaceae) and *Alpinia oxyphylla* Miq. (Zingiberaceae). In a rat model of AD, both ibotenic acid and Aβ_1–42_ were injected into the hippocampus. BYCND was intragastrically administered for 28 days. It was reported that BYCNC significantly decreased the number of Aβ positive neurons in the hippocampus of AD model rats and restored the morphology of neurons inflicted by ibotenic acid and Aβ_1–42_. The BYCND group also showed a shortened escape latency in the Morris water maze. (Chen et al., 2012). However, no followup studies were reported even 10 years after this initial study.

### 3.4 The Danggui Buxue Tang

Containing AR and *Angelica biserrata* (R.H.Shan & C.Q.Yuan) C.Q.Yuan & R.H.Shan (Apiaceae), has been used for over 800 years. In this decoction, the ratio by weight of AR and *Angelica biserrata* (R.H.Shan & C.Q.Yuan) C.Q.Yuan & R.H.Shan (Apiaceae) is 5:1 (Gong et al., 2015). Historically, it is mainly used to treat female menopausal syndromes. Recent studies have also demonstrated its therapeutic effect on cerebrovascular and cardiovascular diseases. It could increase the level of neurotrophic factors and reduce apoptosis induced by Aβ, suggesting its therapeutic potential in managing neurodegenerative disorders, especially AD (Gong et al., 2017; Gong et al., 2019).

### 3.5 The Huangqi Sijunzi Decoction

Is modified from *Sijunzi decoction* [including *Panax ginseng* C.A.Mey. (Araliaceae), *Glycyrrhiza glabra* L. (Fabaceae), *Atractylodes lancea* (Thunb.) DC. (Asteraceae) and *Smilax glabra* Roxb. (Smilacaceae)], with the addition of AR (Cui et al., 2021). Modern pharmacology and clinical studies have shown that HQSJZD has therapeutic potential on AD through multi-ingredient and multi-target mechanisms (Zhang et al., 2021). The active compounds were quercetin, kaempferol, formononetin, isorhamnetin, hederagenin, and calycosin. Their targets were acetylcholinesterase (AChE), prostaglandin-endoperoxide synthase 2 (PTGS2), peroxisome proliferator activated receptor γ (PPARγ), IL-1β, glycogen synthase kinase 3 beta (GSK3B), etc. However, these active compounds were docked with AChE, a target which is less likely to be responsible for cognitive deficits as observed in clinical studies, which show Aricept had limited effect on AD (Birks and Harvey, 2018).

### 3.6 The Bushenyisui decoction

In a rat AD model established by injecting Aβ_1–42_ to bilateral hippocampus, it was found that Bushenyisui decoction treatment for 20 consecutive days increased the level of B-cell lymphoma/leukemia-2 (Bcl-2) and decreased the number of apoptotic cells. As expected, learning and memory deficits were ameliorates as well (Cui et al., 2012). Though this model is different from classical amyloid and tau transgenic mice, it does show learning and memory deficits 5 days after bilateral injections of Aβ_1–42_. Detailed mechanisms underlying the therapeutic effect of Bushenyisui decoction are still under investigation.

Currently, these formulations and patented Chinese medicines containing AR are under investigation to isolate their bioactive compounds and to reveal the underlying mechanisms.

## 4 Potential Mechanisms Underlying the Therapeutic Effect of Bioactive Compounds Extracted From *Astragali radix* on Alzheimer’s Disease

### 4.1 Saponins From *Astragali radix*

It has been reported that there are over 161 saponins isolated from AR, which can be divided into cycloartane and oleanane types (Liu Y et al., 2017). Among them, studies have shown that AS-IV, a quality control marker of AR (Dai et al., 2020), displays a significant therapeutic effect on AD. In this section, we focused on a number of saponins which exhibit anti-AD effects through multiple pathological mechanisms, consisting of preventing the production and aggregation of Aβ, hyperphosphorylation of tau protein, anti-apoptosis, anti-inflammation, promoting the proliferation and differentiation of neural stem cells (NSCs), and so on (Table 1).

**TABLE 1:** The effect and mechanisms of saponins from *Astragali radix* against Alzheimer’s disease.

Saponins	Method	Inducer	Experimental model	Mechanisms	Effects	References
AS-IV	*In vivo*	/	APP/PS1 transgenic mice	PPARγ↑	Inhibit Aβ generation and deposition	Wang et al. (2017)
				BACE1↓		
AS-IV	*In vivo*	AβO	C57BL/6 mice	PPARγ, BDNF, PSD95, SYN, GAP43↑	Inhibit Tau hyperphosphorylation, alleviate synaptic deficits, neuroinflammation, pyroptosis	Wang et al. (2021a)
				NeuN^+^ area↑		
				GFAP, IL-1β, IL-6, TNF-α, NLRP3, cleaved caspase-1↓		
AS-IV	*In vivo*	AβO	ICR mice	ROS, TNF-α, IL-1β, IL-6, NADPH oxidase subunits (gp91phox, p47phox, p22phox, p67phox), Iba-1^+^ area↓	Alleviate cognitive impairment, attenuate neuroinflammation, neuronal damage	Chen et al. (2021)
AS-IV	*In vivo*	Aβ	Sprague-Dawley rats	immature neurons (BrdU+/Tuj1+), Notch-1↑	Promote proliferation and differentiation of NSCs	Hu et al. (2016)
				PS-1↓		
AS-IV	*In vitro*	AβO	HT22 cells	PPARγ, BDNF, cell viability↑	Inhibit apoptosis and cell injury	Wang et al. (2020)
				lactate dehydrogenase release, cleaved caspase-3↓		
AS-IV	*In vivo*	AβO	C57BL/6 mice	Population of healthy cells, PPARγ, BDNF, p-TrkB↑ apoptotic cells, cleaved caspase-3↓	Inhibit neuronal loss, apoptosis	Wang et al. (2020)
AS-IV	*In vitro*	Aβ_25–35_	PC12 cells	SOD↑MDA, ROS, p-p38 MAPK ratio, caspase-3, BIP/GRP78, GADD153/CHOP↓	Attenuate oxidative stress, endoplasmic reticulum stress	Ma and Xiong, (2019)
AST	*In vivo*	AβO	Wistar rats	p-TrkB, p-Akt, p-GSK3β, β-catenin↑	Prevent neuronal degeneration, apoptosis, inhibit Aβ accumulation, alleviate altered microglia polarization and	Wang et al. (2022)
AST	*In vitro*	AβO	Primary cortical neuron	BDNF, TrkB, cathepsin D↑	morphology changes prevent cytotoxicity, apoptosis, attenuate mitochondria distress, synaptotoxicity	Wang et al. (2022)
AST	*In vivo*	/	5xFAD mice	Aβ↓	Inhibit Aβ aggregation	Zhou P et al. (2016)
AST	*In vivo*	Aβ_25–35_	Sprague-Dawley rats	Population of healthy neurons↑ p-Tau↓	Alleviate memory impairments, neuronal degeneration, inhibit cortical Tau hyperphosphorylation	Chang et al. (2016)
AST	*In vitro*	Aβ_25–35_	Primary cortical neuron	Synaptophysin, Cell viability↑	Prevent cytotoxicity, apoptosis, synaptotoxicity, and mitochondrial dysfunction	Chang et al. (2016)
				DNA fragmentation, caspase-3, pTau↓		
Cycloastragenol	*In vivo*	AβO	C57BL/6N mice	Nrf2, HO-1, BDNF, p-TrkB, p-CREB, NeuN, p-ERK, Bcl-2↑	Ameliorate oxidative stress, neurotrophic processes, neuroinflammation, apoptosis	Ikram et al. (2021)
				ROS, LPO, p-JNK, p-p38, Iba-1, GFAP, TNF-α, IL-1β, Bax, Casp-3, Bim, Caspase-3↓		

#### 4.1.1 Inhibiting Aβ Production and Aggregation and Tau Hyperphosphorylation

The amyloid cascade hypothesis pinpoints the central role of deposition of Aβ in the brain parenchyma as an initial event during AD pathogenesis (Ghiso and Frangione, 2002). It is well known that amyloid proteins are produced from the amyloid precursor (APP), a key biomolecule, through cleavage by β-secretase (β-APP- cleaving enzyme-1 (BACE1) at the ectodomain and γ-secretase at intra-membranous sites (Karran et al., 2011; Scheltens et al., 2016; Knopman et al., 2021; Leng and Edison, 2021). In addition, autosomal dominant mutations in presenilin 1 and presenilin 2 also could alter Aβ homeostasis, leading to misfolding of proteins, aggregation, and deposition of Aβ in the brain parenchyma (Jack et al., 2018). This hypothesis posits that the increased level of Aβ results in subsequent pathological changes in AD. Studies have shown that the activity of BACE1 gene promoter could be inhibited by peroxisome proliferator activated receptor γ (PPARγ) agonists (Sastre et al., 2006). Administration of a PPARγ antagonist increased the level of BACE1 and subsequently increased the level of Aβ (Gu et al., 2018). Wang et al. (2017) conducted experiments in cultured SH-SY5Y cells which were transfected with pEGFP-N1-BACE1 and in APP/PS1 mice, both of which were treated with AS-IV. They found that AS-IV *in vitro* activated PPARγ and suppressed the level of BACE1, leading to a significantly decreased level of Aβ. Consistently, AS-IV could significantly suppress the formation of plaques and downregulate the expression of Aβ in the APP/PS1 mouse brain. Interestingly, they also observed that GW9662, an antagonist of PPARγ, could dramatically block the effect of AS-IV. Collectively, these evidences suggested that AS-IV could reduce the production of Aβ through inhibiting BACE1 as a natural PPARγ agonist. In 5xFAD mice, astragaloside was found to decrease the expression of Aβ, but *in vitro* study did not find the level of BACE1 significantly suppressed (Zhou N et al., 2016). In cultured hippocampal neurons, it was found that amyloid β protein fragment 1–42 oligomers (AβO) inhibited the expression of PPARγ and brain derived neurotrophic factor (BDNF), and the phosphorylation of tyrosine receptor kinase B (TrkB). AS-IV significantly reversed the expression of PPARγ and BDNF. Inhibition of PPARγ attenuated the effect of AS-IV on BDNF, suggesting that AS-IV could increase the expression of BDNF. *In vivo* experiments confirmed the positive effect of AS-IV on BDNF and restored the cognitive function of mice injected with AβO (Wang et al., 2020). These studies focused on the production of Aβ, but few studies have examined the effect of saponin from AR on the clearance of Aβ, which may also impact the therapeutic effect of these saponins.

Tau is a microtubule-associated protein that contributes to stabilization of microtubules, axonal outgrowth, and maintaining DNA structure (Leng and Edison, 2021). Pathological alterations of tau might be associated with detachment of tau from microtubules, aggregation of tau, synaptic damage, and consequent cognitive deficits. Recent studies have shown that intrahippocampal infusion of AβO leads to appearance of AD-like phenotypes in mice, such as tau hyperphosphorylation, neuroinflammatory reaction, loss of neurons, synaptic impairment, and fear memory deficits (Jack et al., 2010; Cline et al., 2018). In primary cortical neurons, Chang et al. (2016) observed that astragalosides (AST) could inhibit Aβ_25–35_-induced apoptosis through suppressing tau hyperphosphorylation. This therapeutic effect was reversed by a PI3K inhibitor, suggesting that the PI3K/AKT/GSK-β/β-catenin pathway might be the therapeutic target of astragalosides. Wang et al. (2021a) also found that intrahippocampal infusion of AβO suppressed the expression of PPARγ in the hippocampus of AD model mice. Whereas, AS-IV could reverse the increased production of phosphorylated tau in AβO infused mice, accompanied with improvement of AD-like phenotypes through regulation of PPARγ. Therefore, AS-IV has dual roles in combating AD partially through activating the PPARγ signaling pathway. However, whether this compound decreases tau phosphorylation through other signaling pathways remains to be elucidated.

#### 4.1.2 Anti-Apoptosis

To clarify the anti-apoptosis effect of AST on AD, Chang et al. (2016) established an AD model through infusing amyloid β-protein fragment 25–35 (Aβ_25–35_) into the lateral ventricle of the rat brain. They found that AST inhibited Aβ_25–35_-induced neuronal degeneration and memory deficits in AD model rats. As stated above, AST could inhibit Aβ_25–35_-induced apoptosis through suppressing tau hyperphosphorylation and DNA fragmentation as well as elevating the level of caspase-3. The anti-apoptosis effect of AST weas suppressed by LY294002, an inhibitor of PI3K-dependent protein kinase B (AKT). U0126, an inhibitor of extracellular protein kinase (ERK), displayed the same anti-apoptosis property as AST. These evidences suggested that AST could protect neurons against apoptosis through inhibiting the PI3K/AKT and ERK pathways. Whether AST could suppress apoptosis through increasing the level of anti-apoptosis molecules like Bcl-2 and Bax is unknown.

It is well known that ROS generation elevates cytochrome-c release, activates the expression of caspase proteins, and aggravates mitochondrial swelling at the early stage of apoptosis (Obulesu and Lakshmi, 2014). A study has revealed that the brief opening of mitochondrial permeability transition pore (mPTP) is associated with Aβ-induced apoptosis and ROS release (Zorov et al., 2014). Another study indicated that, in the presence of Aβ_1–42_, AS-IV dramatically suppressed the generation of intracellular ROS and mitochondrial superoxide, blocked the opening of mPTP. Therefore, mitochondrial membrane potential was preserved and the production of ATP restored. The nearly normal supply of ATP maintained the activity of cytochrome c oxidase and suppressed cytochrome c release from mitochondria. Consistent with results of Zorov et al., decreased ROS production by AS-IV significantly reduced the level of cleaved caspase-3, inhibited the expression of Bax, and increased the level of Bcl-2, an anti-apoptosis molecule, in the presence of Aβ_1–42_. These results have proven that AS-IV prevents Aβ-induced apoptosis of SK-N-SH cells by suppressing the opening of mPTP, the production of intracellular ROS, and increasing the level of anti-apoptosis molecules (Sun et al., 2014). *In vivo* study by Ikram et al. (2021) showed similar results. Increased oxidative stress was observed in Aβ injected mice, accompanied by significant neuronal loss and increased levels of Bax, casp-3, and Bim. These indicate that neuronal loss might be attributed to augmented apoptosis. Cycloastragenol, a triterpenoid saponin, significantly improved the cognitive performance of this mouse model and attenuated the changes in apoptosis related molecules as well as neuroinflammation.

During stress, the level of the corticosteroid is increased and this correlates with dementia progression in AD patients. Based on this, Li et al. (2011) injected dexamethasone to 12-month old mice to mimic learning and memory impairment. They found significant increase of casp-3 and -9, increased activities of these proteins, and neuronal apoptosis. Consequently, learning and memory impairment was observed. Extract of AR reversed the behavioural and molecular changes. But which bioactive ingredient was responsible for this therapeutic effect was unknown. It is known that endoplasmic reticulum stress (ERS) is involved in AD progression. In an *in vitro* cell culture model, Aβ_25–35_ significantly increased levels of ERS-specific proteins, BIP/GRP78 and GADD153/CHOP in PC12 cells, as well as casp-3. AS-IV significantly reversed these changes, suggesting that it can regulate ERS induced cell apoptosis (Ma and Xiong, 2019).

#### 4.1.3 Anti-Neuroinflammation

Chen et al. (2021) used AβO-infused mice to explore the anti-neuroinflammation activity of AS-IV. They found that injection of AβO significantly upregulated expression of ROS, IL-1β, IL-6, and TNF-α, which are biomarkers of inflammation. AS-IV could significantly attenuate the increase of these cytokines as shown by the enzyme linked immunosorbent assay. In addition, immunohistochemical staining showed that AS-IV was able to suppress AβO-induced microglial activation and neural injury. Importantly, Western blotting revealed that AS-IV suppressed the up-regulation of NADPH oxidase subunits p22phox, p47phox, p67phox, and gp91phox induced by AβO. Consistent with molecular changes, behavioral tests showed that AS-IV could significantly improve the cognitive performance of mice that received AβO-infusion. Collectively, AS-IV could attenuate AβO-induced AD-like behaviours and molecular changes through suppressing the activation of microglial cells and decreasing the level of NADPH oxidase.

Wang et al. (2021a) found that infusion of AβO dramatically increased the number of astroglia, and this astroglial response was observed in the hippocampus of mice as detected by immunofluorescence staining. AS-IV (20 mg/kg) could effectively decrease the number of astrocytes. Consistently, immunoblotting assay detected that AS-IV suppressed the expression of GFAP in AβO-infused mice. Furthermore, AS-IV significantly decreased pyroptosis, synaptic loss, and the level of proinflammatory cytokines, like IL-1β, IL-6, and TNF-α. Surprisingly, GW9662, an inhibitor of PPARγ, could block the therapeutic effect of AS-IV. These data suggest that the PPARγ signaling pathway is the key target of AS-IV in suppressing the inflammatory reaction as well as other molecular changes in the hippocampus. In another study by Ikram et al. (2021), Aβ was stereotaxically injected into the lateral ventricle of mice to induce the Alzheimer’s disease model, followed by 6 weeks treatment of cycloastragenol, a triterpenoid saponin present in AR. It was found that Aβ injection reduced the number of neurons, levels of BDNF, phosphorylated receptor tropomyosin receptor kinase B (p-TrKB), nuclear factor erythroid 2-related factor 2 (Nrf-2), hemo oxygenase-1 (HO-1), and increased oxidative stress markers, which might be mediated by the MAP kinases (MAPK) as expression levels of p-JNK, p-P-38, p-Erk were increased. Additionally, activation of microglia was increased accompanied by increased levels of proinflammatory cytokines, such as TNF-α, IL-1β, and apoptosis related molecules Bax, casp-3, Bim. Cycloastragenol significantly attenuated these alterations observed in this model mouse and improved the cognitive performance of these mice. The NF-κB mediated signaling pathway is a classical one in producing proinflammatory cytokines in glial cells, whether it is activated by AS-IV or other saponins remains to be investigated.

#### 4.1.4 Promoting Neural Stem Cells Proliferation and Differentiation

Hu et al. (2016) investigated the effect of AS-IV on the proliferation and differentiation of NSCs. In this study, cultured NSCs from the hippocampus of rat embryos were used. NSCs were treated with AS-IV, and then transplanted into the hippocampus of AD model rats. AS-IV could induce NSCs to differentiate into GFAP^+^ and β -tubulin III^+^ cells *in vitro*. In *in vivo* experiments, transplantation of NSCs into AD model rats contributed to the amelioration of AD-like phenotype. AS-IV could augment this beneficial effect. In addition, AS-IV increased the number of β -tubulin III^+^ cells in the hippocampus of AD model rats. Both *in vivo* and *in vitro* experiments showed that AS-IV suppressed presenilin 1 expression, the active subunit of γ-secretase. Paradoxically, AS-IV at both 10^–5^ and 10^–6^ M increased the expression of Notch-1, but AS-IV at 10^–5^ M decreased the level of the Notch intracellular domain-NICD which is cleaved by γ-secretase. In summary, AS-IV facilitated the proliferation and differentiation of NSCs through the Notch signaling pathway and consequently ameliorated AD. Whether AS-IV can regulate NSC proliferation and differentiation through other key regulators is still unknown. Neural stem cell migration is an important step in replenishing dying neurons, this has not been studied with the assistance of saponins.

In another study, A β _25–35_ was injected into the lateral ventricle to induce memory deficits. They found prominent axonal atrophy and synaptic loss in the hippocampus and the cortex. Aqueous extract of AR significantly prevented axonal atrophy, neuronal loss, and increased the outgrowth of axons as well as the formation of synapses in the cortex (Tohda et al., 2006). A study by Cheng et al. (2006) examined the effect of astragaloside at different concentrations on the regeneraton of axons in the periphery. They found that 50 μmol could sigficantly increase the regeneration of axons, and 200 μmol inhibit the growth of axons. This suggests that proper use of bioactive compounds isolated from AR is important.

### 4.2 Flavonoids From *Astragali radix*

Flavonoids are the largest group of polyphenolic compounds isolated from AR, accounting for 4.34% ethanol extract of AR (Xu et al., 2013). Up to now, studies have reveal that several subclasses of flavonoids from been identified from AR, such as quercetin, calycosin, formononetin, sphaemphyside SB, isomucronulatol, formononetin-7-O-β-D-glycoside, methylnissolin, (6aR, l 1aR) 9,10-dimethoxypterocarpan-3-O-β-D-glycoside, 7,2′-dihydroxy-3′,4′-dimethoxy-isoflavane-7-O-β-D-glycoside (Zhang et al., 2012; Wang B et al., 2016). Biological activities of flavonoids vary between each other because of their differences in action mode, and bioavailability (Ullah et al., 2020). The therapeutic effect of well studied compounds were listed in Table 2.

**TABLE 2:** The effect and mechanisms of flavonoids from *Astragali radix* against Alzheimer’s disease.

Flavonoids	Method	Inducer	Experimental model	Mechanisms	Effects	References
Quercetin	*In vivo*	/	APP/PS1 transgenic mice	MMP, ATP, pAMPK172↑	Decrease plaque burden, alleviate mitochondrial dysfunction	Wang et al. (2014)
				Thioavine-S positive compact plaques, ROS production↓		
Quercetin	*In vivo*	/	3xTg-AD mice	C-terminal APP fragments (β), βA 1–40, βA 1–42, PHF-1, AT-8, GFAP, Iba-1↓	Decrease extracellular β-amyloidosis, tauopathy, astrogliosis, microgliosis	Sabogal-Guáqueta et al. (2015)
Quercetin	*In vivo*	Scopolamine	Swiss mice	Population of healthy cells↑	Inhibit apoptosis, attenuate cell injury, and neuroinflammation	Olayinka et al. (2022)
				IL-6, TNF-α, apoptotic cells↓		
Quercetin	*In vitro*	/	CHO cells overexpressing wild-type human APP/SHSY cells	Aβ1-40, Aβ1-42, sAPPβ, APP-CTFβ, BACE-1, NFκB↓	Attenuate neuroinflammation, inhibit Aβ aggregation	Qureshi et al. (2011)
Calycosin	*In vivo*	/	APP/PS1 transgenic mice	Ach, GSH↑ amyloid beta, tau protein, TNF-α, IL-1β, AChE, MDA↓	Inhibit Aβ aggregation and tau hyperphosphorylation, attenuate oxidative stress, and inflammatory response	Song et al. (2017)
FMN	*In vitro*	Aβ_25–35_	HBMEC cells	Cell viability, Nrf2↑	Ameliorate vascular inflammation	Fan et al. (2022)
				VCAM-1, ICAM-1, E-selectin, NFκB activation, nuclear translocation↓		
FMN	*In vivo*	/	APP/PS1 transgenic mice	LRP1, ApoJ↑	Attenuate learning and memory deficit, inhibit Aβ production and increase clearance, alleviate oxidative stress injury, inflammatory response, neuronal cell death, neuronal injury, and improve capillary morphology	Fei et al. (2018)
				Aβ40, Aβ42, APP, RAGE, NF-κB p65, IL-6, TNF-α, IL-1β↓		
FMN	*In vitro*	/	Hypoxic N2a-APP cell	Cell viability, sAβPPα, CTF-α, α-secretase activity, ADAM10, ADAM10 mature/pre-mature ratio↑	Neuroprotection, attenuate cell damage, hypoxia-induced apoptosis, inhibit Aβ aggregation	Sun et al. (2012)
				LDH release, caspase-3↓		
FMN	*In vitro*	Aβ_25–35_	HT22 cells	Cell viability, α-secretase, sAPPα, p-Erα, p-Akt, p-GSK3↑	Inhibit apoptosis, Aβ aggregation, and neuroprotection	Chen et al. (2017)
				β-secretase, Bcl-2, cleaved caspase-3↓		

Quercetin (3,30,40,5,7-pentahydroxyflavone) isolated from AR is a potent flavonoid. It has anti-inflammatory, anti-oxidant, anti-tumor, anti-proliferation properties. ROS are produced in the progression of AD, quercetin can react with these oxygen radicals in the presence of peroxidase to form hydrogen peroxide and semiquinone radical, which are detrimental to proteins, lipid, and DNA of vulnerable neurons (Ademosun et al., 2016). Semiquinone can also react with glutathione to produce stable 6-glutathionyl-Qu to enhance its antioxidant activity (Robaszkiewicz et al., 2007). In addition, it inhibits xanthine oxidase, nitric oxide synthase, and suppresses oxidative stress induced neuronal damage by activating the Nrf-2 antioxidant responsive element, which subsequently increases the production of glutathione (Arredondo et al., 2010). Quercetin has been reported to increase the production of paraoxygenase in astrocytes, neurons, and macrophages, which exerts neuroprotective effects (Boesch-Saadatmandi et al., 2009). An *in vitro* study has shown that this compound suppresses the activity of AchE and consequently increases the level of acetylcholine (Abdalla et al., 2014).

In the meantime, it inhibits secretase enzymes and reduces the production of amyloid proteins (Shimmyo et al., 2008; Khan et al., 2009). This has been confirmed in AD mouse models where quercetin decreases the level of amyloid proteins in the extracellular space, inhibits tau phosphorylation, and ameliorates neuroinflammation evidenced by attenuated microglial and astrocyte activation (Sabogal-Guáqueta et al., 2015). It is known that activated microglial cells can secrete TNF-α, interleukins and interferon-V. Quercetin can not only suppress the expression of inducible nitric oxide synthase, but also decrease levels of proinflammatory cytokines as listed above. One of the target signaling pathways is JNK/Jun (Qureshi et al., 2011). Like saponins isolated from AR, quercetin restores mitochondrial membrane potential, increases ATP production, and inhibits ROS production. Through increasing the level of a key regulator in energy metabolism-AMP activated protein kinase, and the activities of antioxidants, such as superoxide dismutase 2 (SOD-2), quercetin scanvages ROS, decreases the production of amylioid proteins, and accelerates their clearance (Wang et al., 2014).

A recent study has shown that quercetin ameliorates memory impairment in scopolamine treated mice through protecting against neurodegeneration and neuroinflammation (Olayinka et al., 2022). Collectively, quercetin mainly exerts anti-AD effects through the following pathological mechanisms: 1) inhibition of Aβ production, aggregation and tau phosphorylation; 2) inhibition of the activity of AChE; 3) attenuation of oxidative stress and neuroinflammation (Paris et al., 2011; Qureshi et al., 2011; Abdalla et al., 2013; Sabogal-Guáqueta et al., 2015; Costa et al., 2016).

Calycosin is the most abundant one among flavonoids isolated from AR. It has been shown to relieve cognitive deficits elicited by diabetes mellitus, suggesting that it might be effective in improving cognitive performance of AD patients. In an APP/PS1 transgenic mouse study, Song et al. (2017) found that calycosin could reduce the level of Aβ, tau, IL-1β, TNF-α, AChE, and malondialdehyde (MDA) in the hippocampus. Additionally, the increased activity of AchE was diminished by calycosin. However, the neuroprotective effect of calycosin could be blocked by calphostin C, an inhibitor of protein kinase C, suggesting that calycosin ameliorated cognitive deficits in AD model mice through the protein kinase C pathway, including suppressing the production of proinflammatory cytokines, Aβ, tau, and ROS, as well as the activity of AchE.

In another study, similar results were observed. In addition, it was found that calycosin increased the activities of SOD and glutathione peroxidase, attenuating the damage of lipid peroxides to neurons (Yu et al., 2017). Calycosin-7-O-β-D-glucoside has similar pharmacological activities as calycosin, which is reviewed in the study by Li and Huang (2020).

As the most enriched flavonoid isolated from AR, other mechanisms against AD pathogenesis are under investigation. Currently, the majority of studies focus on ischemia-reperfusion injury to the brain. It has been shown that calycosin takes neuroprotective effects against this type of injury through inhibiting the NF-κB signaling pathway and subsequently suppressing neuroinflammation (Wang et al., 2018; Yao et al., 2019), inhibiting autophagy, and increasing the level of Bcl-2, inhibiting the expression of calpain-1 (Guo et al., 2019), and facilitating angiogenesis through increasing levels of vascular endothelial growth factor (VEGF), erythropoietin (EPO), granulocyte-colony stimulating factor (G-CSF), granulocyte macrophage-colony stimulating factor (GM-CSF), and stromal cell-derived factor 1 (SDF-1) (Liu R et al., 2021). These mechanisms might be the same in AD models or patients, which remains to be examined.

Formononetin (FMN) is another flavonoid that has been studied in AD models though more research is focused on its capability of suppressing cancer. In APP/PS1 mice, FMN significantly improved cognitive performance through modulating the function of endothelial cells and the production of amyloid proteins. FMN suppressed the production of amyloid protein through APP processing. It also increased the clearance of amyloid proteins through LRP1. In addition, it suppressed advanced glycation endproducts and RAGE mediated inflammation (Fei et al., 2018), which can indirectly mediate the influx of amyloid proteins into the brain parenchyma. In cultured human brain microvascular endothelial cells, FMN significantly ameliorated inflammation induced by Aβ_25–35_ through activating the Nrf-2 pathway and inhibiting the NF-κB pathway, which consequently decreased the expression of intercellular adhesion molecule 1 (ICAM-1) and the vascular cell adhesion molecule 1 (VCAM-1). Though Nrf2 was increased, its association with Keap1 was attenuated by FMN in a dose dependent manner (Fan et al., 2022). In the high fat diet induced cognitive impairment mouse model, FMN not only reduced body weight of mice, but also improved the cognitive performance through suppressing tau phosphorylation and neuroinflammation induced by high fat diet. These might be due to the activation of the Nrf-2/HO pathway and inhibition of the NF-κB pathway depending on the upregulation of Peroxisome proliferator-activated receptor coactivator-1α (PGC-1α) (Fu et al., 2019). In an *in vitro* model of AD, FMN was shown to protect neurons by increasing the cell viability and decreasing the level of casp-3. The latter effect was proven to be mediated by increasing the activity of α-secretase which produces soluble amyloid proteins (Sun et al., 2012; Chen et al., 2017). In addition, the PI3K/AKT pathway was also activated by FMN. Inhibitors of PI3K or ERα significantly blocked the effect of FMN, indicating that the PI3K/AKT signaling pathway is responsible for the therapeutic effect of FMN (Chen et al., 2017).

Although there are other subtypes of flavonoids isolated from AR, few studies have investigated their individual pharmacological effects. They are applied to the target organs or cultured cells as a total extract. So far, studies have shown that the these flavonoids have similar effects to calycosin, quercetin. In addition, they are known to promote neurogenesis after ischemic stroke (Gao and Li, 2018), but which of them is playing this role is unclear. Therefore, their therapeutic effects are not elucidated here in detail.

### 4.3 Astragalus Polysaccharides

Astragalus polysaccharides (APS), a group of bioactive compounds isolated from AR, possess multifarious effects in the central nervous system, such as anti-inflammatory, antioxidant, immunomodulating properties, which entitle this group of compounds with therapeutic effects on neurodegenerative diseases, particularly AD (Liu Z et al., 2014; Zhao and Dong, 2016; Huang et al., 2017; Qin et al., 2020; Liu X et al., 2021), as shown in Table 3. However, only a small number of studies tested their efficacy in combating AD.

**TABLE 3:** The effect and mechanisms of polysaccharides from *Astragali radix* against AD.

APS	Method	Inducer	Experimental model	Mechanisms	Effects	References
APS	*In vivo*	/	APP/PS1 transgenic mice	Nrf2, SOD, GSH-Px↑	Attenuate oxidative stress, inhibit Aβ accumulation	Qin et al. (2020)
				Keap1, MDA, apoptotic cells, Aβ40 and Aβ42↓		
APS	*In vivo*	/	APP/PS1 transgenic mice	Plaque-associated GFAP, plaque-associated Iba-1↓	Suppress neuroinflammation	Huang et al. (2017)
				Microglia activation to M1		
				TNF-α, iNOS, and IL-12↓		
				Arg-1 and IL- 10↑		
APS	*In vivo*	/	EAE mousemodel	C3, miRNA-155↓	Suppress neuroinflammation	Liu X et al. (2021)

Qin et al. (2020) studied the therapeutic effect of APS on APP/PS1 mice which exhibited increased levels of Aβ, apoptosis, and impairment in spatial learning and memory. In addition, levels of Keap1, cytoplasmic Nrf2, and MDA were dramatically increased in these mice. In contrast, levels of Nrf2 mRNA and intranuclear Nrf2 were remarkably down-regulated, accompanied by decreased levels of SOD and glutathione peroxidase (GSH-Px). APS significantly reversed the expression levels of cytoplasmic Nrf2, intranuclear Nrf2, SOD, MDA, and GSH-Px. Furthermore, APS dramatically suppressed Aβ aggregation and apoptosis, and ameliorated spatial learning and memory deficits of APP/PS1 mice. These suggested that APS might improve the cognitive function of AD model mice through the Nrf2 pathway. This still needs further validation.

In APPswe/PS1dE9 mice, a metabolically stressed AD model, Huang et al. (2017) reported that body weight, levels of leptin and insulin were elevated in this model upon metabolic stress. Similar to other AD mouse models, there are amyloid plaques and activated microglial cells as well as astrocytes surrounding them. APS significantly reversed the alteration of body weight, levels of leptin and insulin, and diminished activated microglial cells and astrocytes around the plaques. However, APS did not remarkably decrease the deposition of Aβ. Therefore, APS could improve behavior performance of APPswe/PS1dE9 mice by counteracting against metabolic stress and ameliorating metabolic stress induced neuroinflammation. In the study by Liu Z et al. (2014), APS was administered to vascular dementia mice. Behavioral tests showed improved memory shown in Morris water maze, which might be explained by the increased level of acetylcholine (an error in this article was noticed, the reliability of this article is doubted). A similar study also found increased NMDAR1 in the hippocampus of the vascular dementia model mouse. Though learning and memory was improved, the level of acetylcholine was decreased (Zhao and Dong, 2016), which arouses our doubt of its fidelity. In an neuroinflammation model, APS inhibited the expression level of miR-155 both *in vivo* and *in vitro* and the polarization of microglia to the M1 phenotype, accompanied by attenuated expression of proinflammatory cytokines, like IL-1α, TNF-α, and C1q (Liu R et al., 2021). Whether APS are involved in other mechanisms against AD needs further investigations.

## 5 Differences Between *Astragalus membranaceus* and *Astragalus mongholicus*

Using LC-UV and MS, it has been shown that the roots of *A. membranaceus* had nearly the same types of flavonoids as those of *A. mongholicus*, but the content of these flavonoids was much lower (1/3) in the roots of *A. membranaceus* (Lin et al., 2000). Genetic studies revealed that the karyotype of *A. membranaceus* was 2n = 2x = 16 = 10 m + 6sm, belonging to type IB, whereas the karyotype of *A. mongolicus* was 2n = 2x = 16 = 8 m + 8sm, belonging to type IC (Yan et al., 2001). *A. membranaceus* from different origins in China had conserved sequences when the internal transcribed spacer 1 (ITS1) of the nuclear ribosomal RNA gene was tested. ITS1 is 100% identical between *A. membranaceus* and *A. mongholicus* (Yip and Kwan, 2006; Liu et al., 2011). Arbitrarily primed polymerase chain reaction (APPCR) demonstrated that samples from Heilongjiang Province were significantly different from samples from non-Heilongjiang regions. Samples from Shanxi and Neimenggu can be differentiated from their unique bands produced from APPCR. Some bands were present only in samples from Neimengu but not in Shanxi, and a number of bands were present only in samples from Shanxi but not from Neimengu and Heilongjiang (Yip and Kwan, 2006). In the study by Zhang et al. (2018) simple sequence repetition (SSR) was used to differentiate between these two varieties. But only 1 SSR was found to be specific for A. membranaceus, but not found in *A. mongholicus*. Single nucleotide polymorphism study showed that ITS/ITS2 can differentiate between these two varieties, with a base C on the 476th spot of ITS sequence of *A. mongholicus* and T of *A. membranaceus* (Zheng et al., 2019).

A study tested seeds of AR samples from different regions and cultivated in Hunyuan under the same conditions. Ultra-performance liquid chromatography (UPLC) coupled with photodiode array detector and evaporative light scattering detectors was used to simultaneously determine four major isoflavonoids and four major saponins. These two AR varieties were distinguished through principal component analysis, but samples of the same species from different regions were unable to be distinguished. These suggest genetic properties appear to be more important for pharmaceutical activities than environmental factors (Liu et al., 2011). Another study on leaves of these two varieties showed 182 variable sites in the chloroplast genome (Wang X et al., 2016). Proteomic analysis of these two varieties revealed that *A. membranaceus* had 717 specific proteins, whereas *A. mongholicus* had 920 specific protein, with 472 shared between them. There were 21 differentially expressed proteins, such as plant pathogenesis-related class 10 (PR-10), nucleoside diaphosphate kinase (NDK-1), glutelin A2, phospholipase D. *A. mongholicus* had 14 proteins highly expressed and *A. membranaceus* had seven highly expressed (Zhao et al., 2020).

Among 47 sapoins identified from AR, 37 were found in *A. membranaceus* with a total content of 19.01 ± 0.87, and 10 in A. mongholicus with a total content of 21.24 ± 1.06 mg/g (Shao et al., 2018). Among 85 flavonoids, 40 were found in A. mongholicus and 31 in *A. membranaceus* (Liu P et al., 2017). Among the flavonoids, the content of calycosin-7-glucoside, ononin, and the total flavonoids was higher in *A. mongholicus* than in *A. membranaceus*, whereas the content of calycosin and formononetin was higher in *A. membranaceus* than in *A. mongholicus* (Zhou et al., 2016). Six alkaloids were also found in *A. mongholicus* (Liu Y et al., 2017). There are studies on the content of other flavonoids and polysaccharides, but their results are not conclusive.

## 6 Perspectives

Even though research into the pathogenesis of AD has made enormous progress, therapeutic strategies targeting the potential pathogenic mechanisms have hardly succeeded (Breijyeh and Karaman, 2020). This raises doubt concerning the prevalent theories of AD.

It is known that decreased cerebral blood flow is the dominant finding on PET scan (which is the optimal diagnostics to differentiate AD from other pathologies) Epidemiological, imaging, and pathologic studies have revealed that AD has the same risk factors as stroke, and vascular, especially microvascular changes are prominent in the brain tissue of AD patients (Iadecola 2013). But few drugs have been developed to restore the blood flow to the brain. A recent study found that sildenafil, a drug used to dilate blood vessels, was associated with 69% reduced risk of AD (Fang et al., 2021). This suggests that restoration of the cerebral blood flow may prevent cognitive decline.

Studies in the past decade have also found that pericytes surrounding the capillaries play an important role in AD (Nation et al., 2019; Nortley et al., 2019). These cells can regulate the cerebral blood flow to the microcirculation and transform to other types of cells in diseased conditions. Hence, developing therapeutic strategies targeting the pericytes might be a promising direction for AD research.

Currently, there are a myriad of compounds present in the natural products. Network pharmacology has the capability of matching these compounds, with molecular targets in the brains of AD patients or rodent models. It is very likely that potential therapeutic agents will be found using these techniques.

## Glossary

Aβ: amyloid-β
AβO: Aβ (1–42) oligomers
AChE: acetylcholinesterase
AD: Alzheimer’s disease
AKT: phosphoinositide 3-kinase (PI3K)-dependent protein kinase B
APP: the amyloid precursor
APPCR: arbitrarily primed polymerase chain reaction
APS: astragalus polysaccharide
AR: *Astragali radix*
AS-II: astragaloside II
AS-IV: astragaloside Ⅳ
AST: Astragalosides
BACE1: β-APP-cleaving enzyme-1
Bax: the BCL-2-associated X protein
Bim: Bcl-2 interacting mediator of cell death
BIP/GRP78: immunoglobulin heavy chain binding protein BiP/78-kDa glucose-regulated protein
BDNF: brain derived neurotrophic factor
BHD: Buyang Huanwu decoction
BYCNC: Buyuan Congnao decoction
ERα: estrogen receptor alpha
ERS: endoplasmic reticulum stress
EPO: erythropoietin
GADD153/CHOP: growth arrest and DNA damage-inducible gene 153/C/EBP homologous protein
G-CSF: granulocyte-colony stimulating factor
GM-CSF: granulocyte macrophage-colony stimulating factor
GSH-Px: glutathione peroxidase
GSK3B: glycogen synthase kinase 3 beta
HO-1: hemo oxygenase-1
HPLC: high performance liquid chromatography
HQSJZD: Huangqi Sijunzi decoction
ICAM-1: intercellular adhesion molecule 1
IL-1β: interleukin-1β
IL-6: interleukin-6
ITS1: internal transcribed spacer 1
JNK: c-jun-NH2-terminal kinase
Keap1: Kelch-like ECH-associated protein 1
LRP1: lipoprotein receptor-related protein 1
MAPK: mitogen-activated protein kinase
MDA: malondialdehyde
mPTP: mitochondrial permeability transition pore
NADPH: nicotinamide adenine dinucleotide phosphate
NDK-1: nucleoside diaphosphate kinase
NSCs: neural stem cells
NXTC: Naoxintong capsule
NF-kB: nuclear factor-kappa-B
Nrf-2: nuclear factor erythroid 2-related factor 2
PGC-1α: Peroxisome proliferator-activated receptor coactivator-1α
PI3K: phosphoinositide 3-kinase
PR-10: pathogenesis-related class 10
p-TrKB: phosphorylated receptor tropomyosin receptor kinase B
PPARγ: peroxisome proliferator activated receptor γ
PTGS2: prostaglandin-endoperoxide synthase 2
RAGE: receptor for advanced glycation endproducts
ROS: reactive oxygen species
SDF-1: stromal cell-derived factor 1
SOD-2: superoxide dismutase 2
SP: senile plaques
SSR: simple sequence repetition
TCM: Traditional Chinese Medicine
TNF-α: tumor necrosis factor α
TrkB: tyrosine receptor kinase B
UPLC-MS/MS: ultra-performance liquid chromatography-tandem mass spectrometry
VCAM-1: vascular cellular adhesion molecule-1
VEGF: vascular endothelial growth factor
